# Biomimetic Gradient Microporous Scaffold with a Triply Periodic Minimal Surface Enhances Osseointegration by Modulating Macrophage Polarization

**DOI:** 10.34133/bmr.0266

**Published:** 2025-10-17

**Authors:** Li Liu, Hao Chen, Aobo Zhang, Weilong Zhang, Yang Liu, Yongyue Li, Le Gao, Qing Han, Bingpeng Chen, Jincheng Wang

**Affiliations:** ^1^ Department of Orthopedic Surgery, The Second Hospital of Jilin University, Changchun 130000, Jilin, China.; ^2^ Department of Orthopedics, Hebei Province Cangzhou Hospital of Integrated Traditional and Western Medicine, Cangzhou No. 2 Hospital, Cangzhou 061000, Hebei, China.; ^3^ Hebei Key Laboratory of Integrated Traditional and Western Medicine in Osteoarthrosis Research, Cangzhou 061000, Hebei, China.

## Abstract

Bionically designed gradient microporous scaffolds have garnered considerable attention in orthopedics and bone tissue engineering for their ability to replicate the gradual pore transition from cortical to trabecular bone, thereby integrating mechanical strength with biological functionality essential for bone regeneration. However, the immune-inflammatory response induced by biomaterial implantation can impair osseointegration, potentially leading to chronic inflammation or implant failure. To address this limitation, the present study utilized selective laser melting to fabricate a biomimetic gradient microporous titanium alloy scaffold based on a triply periodic minimal surface architecture (pore size: 650 to 350 μm), with solid nonporous scaffolds (0 μm) and uniform microporous scaffolds (500 μm) serving as controls. The study comprehensively evaluated the role of the gradient scaffold in both osseointegration and immune modulation. Mechanical testing confirmed that the gradient scaffold possessed an elastic modulus well matched to that of bone tissue, thereby mitigating stress shielding. In vitro assays revealed that, relative to the control scaffolds, the gradient scaffold more effectively promoted macrophage polarization toward the M2 phenotype while enhancing the osteogenic differentiation capacity of bone marrow mesenchymal stem cells. Subsequent in vivo experiments demonstrated that the gradient microporous titanium alloy scaffold attenuated local inflammatory responses and facilitated new bone formation. Collectively, these findings provide compelling evidence for the dual role of titanium alloy gradient biomimetic microporous structures in immune regulation and osseointegration, offering critical insights for the optimization of scaffold designs in bone tissue regeneration.

## Introduction

Bone defects arising from trauma, infection, tumors, or other pathological factors often necessitate bone grafting or artificial implants for structural reconstruction [1,2]. However, the clinical application of autologous and allogeneic bone grafts is constrained by donor site morbidity, limited availability, immune rejection, and potential disease transmission [3,4]. In recent years, bone tissue engineering scaffolds have attracted considerable attention for their customizable architectures and biomimetic repair potential. Among these, titanium alloy implants are extensively used in clinical practice owing to their excellent biocompatibility, high mechanical strength, and superior corrosion resistance [5,6]. Structural optimization of metallic implants—specifically in pore size, porosity, and geometry—has been shown to markedly enhance osseointegration [7–9]. Microporous architectures not only lower the elastic modulus to mitigate stress shielding but also facilitate cell adhesion, proliferation, nutrient diffusion, and bone ingrowth via interconnected pore networks [10,11]. Pore size plays a decisive role: smaller pores (<100 μm) support cell adhesion and differentiation but, if too small, may cause cell aggregation, impair migration, and restrict nutrient exchange [12]. Larger pores (>300 μm) tend to favor new bone formation and angiogenesis [13]; however, pores exceeding 1,000 μm may compromise structural integrity, slow bone growth, and weaken implant stability [12]. Current research generally agrees that a pore size range of 300 to 700 μm provides an optimal balance between mechanical performance and biological function, creating an ideal microenvironment for promoting osseointegration [8,12,14,15]. Moreover, gradient microporous scaffolds—such as those with pore sizes decreasing from 650 to 350 μm—have been reported to further enhance osseointegration compared to homogeneous designs [16].

Despite these advances, immune-inflammatory responses triggered by biomaterial implantation can profoundly influence osseointegration outcomes. Dysregulated immune activity may lead to chronic inflammation, fibrous encapsulation, and implant failure, thereby undermining bone–implant integration and tissue repair [17]. Consequently, tailoring material properties to modulate the immune microenvironment has emerged as a critical strategy for enhancing osseointegration. Importantly, pore architecture not only governs mechanical and osteogenic performance but also regulates immune responses, which in turn impact bone regeneration. Macrophages are key mediators within this immune milieu, capable of adopting distinct phenotypes in response to local cues. Classically activated M1 macrophages participate in the initial inflammatory phase, contributing to pathogen clearance and inflammation resolution, yet prolonged M1 dominance can exacerbate tissue damage and impair bone healing [18]. Alternatively activated M2 macrophages exert anti-inflammatory effects and promote tissue repair, with timely M1-to-M2 transition being pivotal for effective bone regeneration [19]. Macrophage polarization has been shown to correlate strongly with scaffold pore characteristics. For example, in a murine subcutaneous implantation model using gelatin–hyaluronic acid scaffolds, Jiang et al. [20] found that 30-μm pores favored M1 polarization, whereas 80-μm pores promoted M2 polarization. Similarly, Takabatake et al. [7], employing tricalcium phosphate scaffolds in a rat femoral muscle model, observed that 300-μm pores induced pronounced M1 infiltration, while 500-μm pores favored M2 recruitment. In 3-dimensionally printed polycaprolactone/polyethylene glycol/hydroxyapatite scaffolds, 600-μm pores were associated with increased M2 polarization, enhanced angiogenesis, and improved osteogenesis [21]. While these findings underscore the role of pore size in immune modulation and bone regeneration, existing studies have predominantly examined homogeneous pore structures. The mechanisms by which titanium alloy gradient microporous structures facilitate osteogenesis through immune regulation remain largely unexplored, warranting further investigation.

Natural bone exhibits a porosity gradient from cortical to trabecular regions, where the higher porosity of the inner layer facilitates nutrient transport and the lower porosity of the outer layer provides mechanical strength [22]. Most conventional homogeneous microporous scaffolds fail to replicate this gradient architecture, resulting in suboptimal mechanical compatibility and limited biological function [23]. Recent advances in biomimetic gradient microporous scaffold design address this limitation by continuously varying pore size or porosity, thereby improving both mechanical performance and permeability [24]. Their irregular structural geometry also enhances cell migration, proliferation, and differentiation, supporting osseointegration [25]. Among emerging architectures, the triply periodic minimal surface (TPMS) structure—a continuous 3-dimensional (3D) surface with infinite periodicity and zero mean curvature—offers distinct advantages over traditional lattice, polyhedral, and truss designs. TPMS structures provide tunable parametric design, a high load-bearing capacity, and a large specific surface area [23]. In microporous scaffold applications, they deliver sufficient mechanical strength for bone regeneration, while their beam-like geometry mimics native bone, facilitating oxygen and nutrient transport and promoting integration with surrounding tissue [26,27]. TPMS scaffolds also create a favorable osteogenic and immunoregulatory microenvironment [28]. Comparative studies of common TPMS variants—primitive (P), gyroid (G), and diamond (D)—indicate that TPMS-P structures excel in mechanical performance, permeability, and osteogenesis [23]. However, their influence on the immune microenvironment remains poorly understood. Moreover, most existing research has focused on homogeneous TPMS scaffolds, leaving the immunomodulatory potential of biomimetic gradient TPMS structures largely unexplored.

**Fig. 1.**
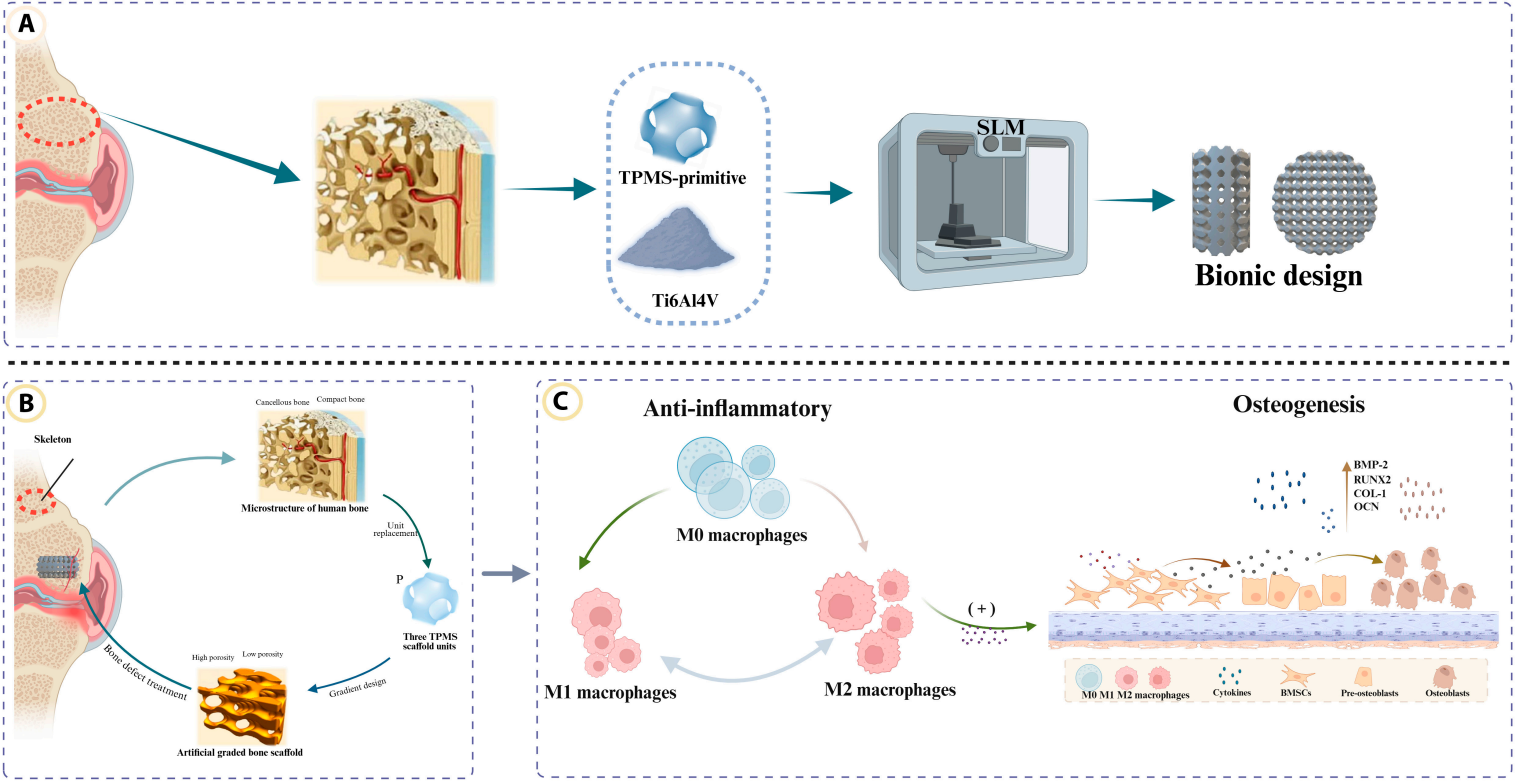
(A to C) Schematic illustration of biomimetic gradient microporous titanium alloy scaffolds’ fabrication and their immunomodulatory and osseointegration effects in vitro and in vivo. TPMS, triply periodic minimal surface; SLM, selective laser melting; BMP-2, bone morphogenetic protein-2; RUNX2, Runt-related transcription factor 2; COL-1, type 1 collagen; OCN, osteocalcin; BMSCs, bone marrow mesenchymal stem cells.

The present study employed selective laser melting (SLM) to fabricate biomimetic gradient microporous titanium alloy scaffolds based on the TPMS-P architecture. Through combined in vitro and in vivo analyses, we systematically evaluated their effects on macrophage polarization and osseointegration. Comparisons with homogeneous microporous and solid scaffolds allowed us to assess osseointegration potential and reveal possible immune-regulatory benefits. The results aim to provide a theoretical foundation for the structural optimization and clinical translation of bone-targeted immunomodulatory biomaterials (Fig. 1).

## Materials and Methods

### Characterization of the scaffolds

#### Fabrication of scaffolds

Three types of scaffolds were designed in this study, including solid scaffolds, uniform microporous scaffolds, and gradient microporous scaffolds, the latter 2 fabricated based on the TPMS-P architecture. To minimize the impact of porosity, the 2 groups of scaffolds were designed to have similar porosity values (uniform: 55.24%; gradient: 57.25%). Cylindrical scaffolds (φ5 mm × L8 mm) were designed for in vivo experiments and compressive mechanical testing (Fig. S1), while disk-shaped scaffolds (φ10 mm × L2 mm) were designed for in vitro experiments (Fig. 1A). The titanium alloy scaffolds used in this study were designed using the computer-aided design software Rhinoceros 6 and the Grasshopper plug-in (McNeel, Seattle, USA). The designs of both the uniform microporous and gradient biomimetic microporous titanium alloy scaffolds were based on the following formula:

$$\cos\left(x\right)+\cos\left(y\right)+\cos\left(z\right)=0$$ (1)

The experimental samples were fabricated using the SLM system BLT-A3200. Ti6Al4V powder with a particle size range of 20 to 50 μm was used as the raw material. The designed titanium alloy models for each group were first input into the metal 3D printer in STL file format. The 3D metal printer then employed SLM technology to fabricate microporous scaffolds from the Ti6Al4V powder. The processing parameters were set as follows: laser power, 150 W; laser scanning speed, 1,200 mm/s; powder layer thickness, 0.03 mm; and hatch distance, 0.1 mm. After the SLM process, the samples were heat-treated in argon gas at 800 °C for 2 h. Afterward, the samples were thoroughly cleaned using anhydrous ethanol and double-distilled water via ultrasonic cleaning to remove any unmelted powder from the surface. Prior to the experimental procedures, the samples were sterilized in a high-pressure autoclave (120 °C, 20 min) and then placed in a constant-temperature oven at 60 °C. Prior to the experiments, the scaffolds were exposed to ultraviolet light for 30 min.

#### Scanning electron microscopy

The microstructure of the scaffolds was examined, and energy-dispersive x-ray spectroscopy (EDS) was performed using a scanning electron microscope (SU-8100, Hitachi, Japan). The surface chemical composition of the scaffolds was further characterized by EDS. Micro-computed tomography (micro-CT) scanning was conducted on 3 samples of each microporous scaffold type, and 3D reconstruction was performed using commercial software (CTVol).

The porosity and pore size of the uniform and gradient microporous scaffolds were calculated. Porosity was defined as the ratio of the volume of pores to the total volume, while pore size was defined as the diameter of the pores within the microporous scaffolds. The surface laser intensity of the samples was measured using a VK-150K laser microscope (KEYENCE, Japan) with a wavelength of 658 nm. The captured laser intensity data were processed using VK-H1XAC software to reconstruct 3D images and analyze surface morphology data.

#### Hydrophilicity test

The water contact angle was measured using the drop test with KRUSS DSA100 (Germany) to evaluate the hydrophilicity of different scaffolds. Three samples were taken from each group, and the water contact angle is expressed as mean ± standard deviation.

#### Compression testing of scaffolds

Compression tests were carried out using a universal testing machine (Autograph CSS-44100, Changchun Institute of Testing Machines, Changchun, China) at a speed of 1 mm/min. Three replicates (*n* = 3) were tested for each group. During the compression process, only vertical displacement was allowed, and the compressive force and displacement were recorded. Stress–strain curves were plotted using Origin (OriginPro 2022, OriginLab, USA), and the elastic modulus of each sample was determined by calculating the slope of the linear portion of the stress–strain curve.

### In vitro testing

#### Cell culture

Bone marrow mesenchymal stem cells (BMSCs) were isolated from the femoral bone marrow of 3-week-old male Sprague–Dawley rats. The cells were cultured in a basic medium supplemented with 10% fetal bovine serum and 1% penicillin/streptomycin. The medium was replaced 24 h after initial seeding and subsequently every 72 h. The cells were maintained at 37 °C in a 5% CO_2_ incubator. Once the cells reached the third passage, they were used for subsequent experiments. For osteogenic induction, β-glycerophosphate (10 mmol/l), dexamethasone (100 nM), and ascorbic acid (0.05 mmol/l) were added to the culture medium to form the osteogenic induction medium.

The mouse-derived macrophage cell line Raw 264.7 was purchased from the Chinese Academy of Sciences Cell Bank and was used to evaluate the inflammatory response to the samples. The cells were cultured in Dulbecco’s modified Eagle medium (Gibco, Grand Island, NY, USA) containing 10% fetal bovine serum at 37 °C in a humidified incubator with 5% CO_2_.

#### Cytotoxicity and proliferation on samples

The proliferation of BMSCs and Raw 264.7 cells on different samples was assessed using Cell Counting Kit-8 (CCK-8, Bioss, USA). Cells were seeded onto the scaffolds and cultured in 24-well plates for 1, 3, and 5 d. After the incubation periods, the culture medium was aspirated, and the scaffolds were transferred to new 24-well plates. The scaffolds were then incubated in a culture medium containing 10% CCK-8 at 37 °C for 2 h. The optical density (OD) values of each group were measured at 450 nm using an automated enzyme-labeling instrument (Bio-Rad, CA, USA).

The live/dead assay was used to assess the cytotoxicity of different scaffolds to BMSCs and Raw 264.7 cells. After culturing the cells with the scaffolds for 1 and 3 d, the cells were stained using a live/dead assay kit (BestBio, China). Images of the live/dead staining were collected using a fluorescence microscope (Olympus, Japan).

#### Cytoskeleton assembly

Scaffolds from different groups were placed in 24-well plates, and BMSCs were seeded onto each scaffold at a density of 5 × 10^4^ cells per scaffold. After 3 d of culture, the medium was discarded, and the cells were rinsed 3 times with phosphate-buffered saline (PBS). The cells were then fixed with 4% paraformaldehyde for 30 min, followed by 3 PBS washes. Permeabilization was performed with 0.2% Triton X-100 for 15 min, after which the cells were again washed 3 times with PBS. The actin cytoskeleton was stained with fluorescein isothiocyanate-labeled phalloidin (Yeasen, China) for 30 min, and the nuclei were counterstained with 2-(4-amidinophenyl)-6-indolecarbamidine dihydrochloride (DAPI) solution for 5 min. Excess DAPI was removed by washing with PBS, and images were acquired using an immunofluorescence microscope (Olympus, Japan).

#### Osteogenic activity of BMSCs cultured on the scaffolds

##### Alkaline phosphatase staining and quantification

Different groups of scaffolds were placed in 24-well plates, and BMSCs were seeded onto the scaffolds at a density of 5 × 10^4^ cells per scaffold. The cells were cultured in a 37 °C, 5% CO_2_-humidified incubator. After 2 d of culture in basic medium, the medium was replaced with osteogenic induction medium. The osteogenic medium was refreshed every 2 d. After 7 and 14 d of osteogenic induction, alkaline phosphatase (ALP) staining was performed using a BCIP/NBT ALP colorimetric kit (Beyotime, China). The samples were rinsed with PBS, fixed with 4% paraformaldehyde for 10 min, and subsequently stained in the dark at room temperature. The staining results were examined using a stereomicroscope (BX51TF, Olympus, Japan). ALP activity was quantitatively assessed with a commercial ALP assay kit (Beyotime, China). To standardize the results, the total protein content was determined using a bicinchoninic acid protein assay kit (Beyotime, China), and ALP activity was expressed relative to total protein levels.

##### Alizarin Red S staining and quantification

BMSCs were seeded and cultured as described in the “Alkaline phosphatase staining and quantification” section. After 14 d of osteogenic induction, Alizarin Red S (ARS) staining was performed to assess the formation of mineralized nodules. Briefly, the samples were washed 3 times with PBS, fixed in 4% paraformaldehyde for 10 min at room temperature, and stained with ARS solution (Beyotime, China) at 37 °C for 30 min. After staining, the samples were washed with distilled water to remove unbound dye. Following observation, the samples were transferred to new 24-well plates. Quantification was performed by dissolving the stain with a 10% cetylpyridinium chloride solution, and the OD of each group was measured at 562 nm using a microplate reader.

##### Real-time quantitative PCR for osteogenesis-related genes

Quantitative polymerase chain reaction (PCR) was performed to evaluate the osteogenic differentiation of BMSCs on different scaffolds. After 7 and 14 d of osteogenic induction, total RNA was extracted using TRIzol reagent (Invitrogen, Carlsbad, CA, USA) and reverse-transcribed into complementary DNA. Real-time quantitative PCR (RT-qPCR) was performed using an SYBR Green detection kit (GeneCopoeia Inc., USA) on a LightCycler 480 machine (Roche Co., Ltd., Switzerland). Osteogenesis-related gene markers, including bone morphogenetic protein-2 (BMP-2), Runt-related transcription factor 2 (RUNX2), osteocalcin (OCN), and type 1 collagen (COL-1), were analyzed, with glyceraldehyde-3-phosphate dehydrogenase (GAPDH) used as the housekeeping gene. Relative gene expression levels were calculated using the comparative Ct method (2^−ΔΔCt^). The forward and reverse primer sequences used in this study are listed in Table S1.

#### Preparation of the conditioned medium

##### Culturing Raw 264.7 cells and conditioned medium preparation

Raw 264.7 cells were seeded onto the various samples in 24-well plates at a density of 4 × 10^4^ cells per well and cultured for 4 d. The culture supernatant was collected, centrifuged, and mixed with the osteogenic induction medium at a 1:2 ratio to generate a conditioned medium. BMSCs were seeded onto 24-well plates at a density of 4 × 10^4^ cells per well. On day 3, the growth medium was replaced with the conditioned medium, and thereafter, the medium was changed every 2 d.

#### Macrophage response to the scaffold surface

##### ELISA and RT-qPCR analysis of macrophage polarization

After 4 d of incubation with the samples, the Raw 264.7 cell culture supernatant was collected and centrifuged. The concentrations of tumor necrosis factor-α (TNF-α) and arginase 1 (Arg-1) in the supernatant were measured using an enzyme-linked immunosorbent assay (ELISA) kit (Jingmei, China), according to the manufacturer’s instructions. Additionally, RT-qPCR was performed to assess the expression levels of M1 macrophage markers (inducible nitric oxide synthase [iNOS] and TNF-α) and M2 macrophage markers (Arg-1 and interleukin-10 [IL-10]). After incubating Raw 264.7 cells in the culture medium for 4 d, RNA was extracted and reverse-transcribed into complementary DNA. RT-qPCR analysis was performed using SYBR Green detection reagents, with GAPDH as the housekeeping gene. All experiments were performed in triplicate. Relative gene expression levels were calculated using the comparative Ct method (2^−ΔΔCt^). The forward and reverse primer sequences used in this study are shown in Table S2.

#### Osteogenic differentiation evaluation of the Raw 264.7-conditioned medium

ALP staining was performed on days 7 and 14 of osteogenic induction, while ARS staining was conducted on day 14. Subsequently, RT-qPCR analysis was carried out following cell extraction. The evaluation of osteogenic differentiation in the Raw 264.7-conditioned medium was conducted in accordance with the procedures used to assess the osteogenic activity of BMSCs cultured on scaffolds.

### In vivo testing

#### Subcutaneous implantation in vivo

All in vivo animal experiments were conducted in accordance with the guidelines of the Chinese Animal Protection and Utilization Committee for the care and use of laboratory animals. The animal experiments were approved by the Animal Ethics Committee of the College of Basic Medical Sciences, Jilin University, and were performed following the committee’s ethical guidelines. Subcutaneous implantation models were established in 8-week-old male Sprague–Dawley rats. All implanted scaffolds were sterilized by high-pressure autoclaving, and the surgeries were performed under sterile conditions. Prior to implantation, the rats were anesthetized with isoflurane, the dorsal skin was shaved and disinfected, and a small incision was made at the center of each rat’s back. Scaffolds from different groups were implanted into subcutaneous pockets, and the incisions were carefully sutured. All experimental animals recovered well postsurgery, with no complications, and the wounds healed properly. On day 14 postimplantation, the rats were euthanized, and skin tissue around the scaffold implantation site was collected for immunofluorescence staining.

#### Immunofluorescence staining

Immunofluorescence staining was performed on the subcutaneous tissue surrounding the scaffolds in rats to analyze macrophage polarization. Briefly, after standard dewaxing of the samples, antigen retrieval was performed using an antigen retrieval kit, and the slides were then microwaved at the recommended power for 20 min. After natural cooling, the slides were washed with PBS on a shaker to remove residual staining. The samples were subsequently blocked to prevent nonspecific binding of the primary antibody. The slides were incubated overnight at 4 °C with the primary antibodies. Rabbit anti-C-C motif chemokine receptor 7 (anti-CCR7) antibody (M1 marker, Boster, China) and rabbit anti-mannose receptor (anti-CD206) antibody (M2 marker, Boster, China) were used to stain the samples, as these are well-known markers for M1 and M2 macrophage phenotypes, respectively. After washing with PBS, the slides were incubated with fluorescently labeled secondary antibodies and stained with DAPI for nuclear visualization. Finally, images were captured using a fluorescence microscope (Olympus, Japan).

#### Distal femoral bone defect in vivo

To compare the osteogenic potential of different scaffold groups and the influence of the surrounding bone tissue on macrophage polarization, an in vivo experiment was conducted with 18 adult male New Zealand rabbits (2 to 2.5 kg), divided into 3 groups (*n* = 6). The rabbits were anesthetized via intravenous injection of sodium pentobarbital. The fur around the femur and knee joint was shaved, and the skin was thoroughly disinfected. Local anesthesia was achieved with lidocaine. A skin incision was made, and the skin, the fascia, and other tissues were dissected to expose the lateral femoral condyle. A bone drill was used to create a critical-size cylindrical bone defect with a diameter of approximately 5 mm and a depth of 8 mm in the distal femur. The cylindrical scaffolds were then implanted into the femoral condyle defects. The incisions were closed layer by layer using absorbable sutures. To prevent infection postsurgery, the rabbits were intramuscularly injected with penicillin for 3 d. At the designated time points, the rabbits were euthanized via CO_2_ asphyxiation, and tissue samples were collected. The experimental procedure is shown in Fig. S2.

#### Micro-CT analysis

After obtaining femoral samples, they were fixed in 4% paraformaldehyde. Micro-CT scanning was conducted to assess the effect of the different scaffold groups on osseointegration. The samples were placed in the imaging system for radiological analysis; 3D reconstructions of the acquired images were generated using multimodal 3D visualization software (Skyscan 1076 Scanner, Bruker Micro-CT, NV, Kontich, Belgium). Image analysis was performed using CT analyzer software (CT Analyzer 1.17.7.2, Kontich, Belgium). The area surrounding the implanted scaffold was defined as the region of interest. Parameters such as bone volume (BV), total volume (TV), bone surface area (BS), and trabecular thickness (Tb.Th) were quantified to assess osseointegration.

#### Histological analysis

Rabbit femur samples were fixed in 4% paraformaldehyde. Subsequently, the samples were subjected to a gradient dehydration process using ethanol (70%, 75%, 80%, 85%, 90%, and 95% ethanol) for 2 h each. The samples were then embedded and sectioned. The embedded femur specimens were sectioned to a thickness of approximately 40 μm using a hard-tissue-cutting machine (EXAKT 300CP, EXAKT, Germany). The sections were stained with methylene blue and acidic fuchsin to assess bone tissue regeneration and mineralization in the different groups. The stained sections were photographed and observed under an optical microscope (Eclipse E100, Nikon, Japan).

#### Immunohistochemistry staining

The femur samples, obtained 1 month postsurgery, were decalcified with 10% EDTA for 2 months. After decalcification, the implants were removed from the femur, and surrounding tissues were processed for immunohistochemistry (IHC) staining. Briefly, tissue sections were deparaffinized and rehydrated through a series of decreasing ethanol concentrations, followed by antigen retrieval and hydrogen peroxide incubation. Nonspecific binding was blocked by incubating with blocking buffer at room temperature. The sections were then incubated overnight at 4 °C with primary antibodies for RUNX2 (ABclonal, China), BMP-2 (ABclonal, China), Arg-1 (ABclonal, China), and CCR7 (ABclonal, China). After incubation with primary antibodies, the sections were incubated with secondary antibodies. Immunoreactivity was visualized using a diaminobenzidine chromogenic substrate. The sections were counterstained with hematoxylin, dehydrated, cleared, and mounted. After staining, histological images were captured using an optical microscope (Olympus Corporation, Tokyo, Japan) and analyzed with the ImageJ software (National Institutes of Health, Bethesda, MD, USA).

### Statistical analysis

All experimental results are expressed as mean ± standard deviation. One-way analysis of variance was performed in GraphPad Prism 9, followed by Tukey’s post hoc test, with *P* < 0.05 considered statistically significant.

## Results

### Design, fabrication, and characterization of Ti6Al4V scaffolds

Gradient microporous, uniform microporous, and solid nonporous titanium alloy scaffolds were manufactured via SLM. Key parameters—including porosity and pore size—were characterized using scanning electron microscopy (SEM) and micro-CT. The uniform microporous scaffold had a pore size of 500 ± 8 μm and a porosity of 56.96% ± 0.3%, while the gradient microporous scaffold had a maximum pore size of 650 ± 12 μm, a minimum pore size of 350 ± 6 μm, and a porosity of 58.10% ± 0.4%. The comparable porosity ensured that pore size differences were the primary determinants of osteogenic and immunomodulatory effects in the study.

**Fig. 2.**
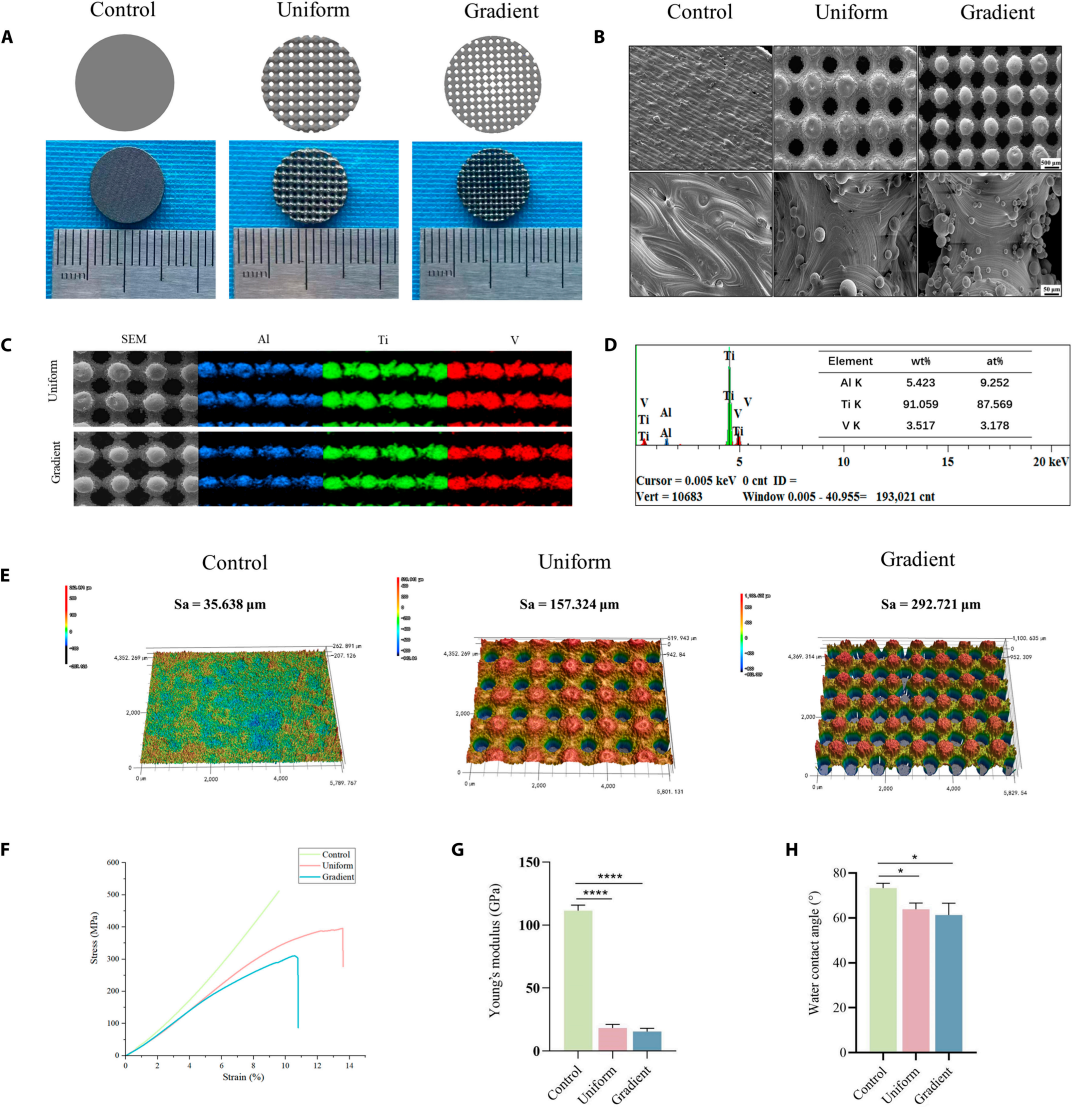
Structural characterization and mechanical properties of the scaffolds. (A) Models and macroscopic images of the scaffolds in vitro. (B) Microscopic scanning electron microscopy (SEM) images of the scaffolds. (C) Energy-dispersive x-ray spectroscopy (EDS) elemental distribution maps for the uniform and gradient scaffolds. (D) EDS analysis showing the percentage of surface elemental content of the scaffold. (E) Reconstruction of the 3-dimensional surfaces of different scaffolds. (F) Stress–strain curves for each scaffold group. (G) Diagram of the elastic modulus for each scaffold group. (H) Hydrophilicity of the scaffolds. Data are presented as mean ± SD (*n* = 3). * indicates significant differences (*P < 0.05; **P < 0.01; ***P < 0.001; ****P < 0.0001).

Macroscopic images confirmed that the printed scaffolds matched the intended design (Fig. 2A and B). SEM imaging revealed residual metal particles from SLM-processed Ti6Al4V powder, creating a surface that was not entirely smooth. Both microporous scaffold types displayed well-connected struts with no visible deformation or fractures. The continuous micropore network supports effective circulation of cells and nutrients postimplantation [29]. EDS identified Ti, Al, and V as the primary surface elements, consistent with the Ti6Al4V composition, and detected no extraneous elemental contamination (Fig. 2C and D).

Three-dimensional imaging at ×50 magnification (Fig. 2E) was used to assess surface morphology. The arithmetic mean surface roughness (Sa), a key determinant of cell–surface interactions, varied significantly among scaffold types. The solid scaffold had the smoothest surface, the uniform microporous scaffold showed a higher Sa, and the gradient microporous scaffold exhibited the highest Sa (292.721 μm), demonstrating the important role of structural design in surface characteristics.

Static compression tests showed that solid scaffolds had the highest elastic modulus (110.0 ± 1.81 GPa). Between the microporous groups, stress–strain curve analysis (Fig. 2F) revealed substantial differences in stress and fracture limits, although elastic moduli were comparable: 17.34 ± 1.29 GPa for the uniform scaffold and 16.05 ± 1.05 GPa for the gradient scaffold (Fig. 2G). Given that natural bone exhibits an elastic modulus of approximately 3 to 30 GPa, both microporous scaffolds closely matched bone stiffness, thereby reducing stress shielding and supporting osseointegration.

Water contact angle measurements showed the solid scaffold to be the least hydrophilic, the uniform microporous scaffold moderately hydrophilic, and the gradient microporous scaffold the most hydrophilic (Fig. 2H). Enhanced hydrophilicity in the gradient design facilitated cell adhesion, nutrient uptake, and protein adsorption, creating a favorable microenvironment for bone healing and accelerating osseointegration [30].

### Biocompatibility evaluation

In this study, flow cytometry was used to detect cell surface markers and confirm the identity of the collected cells as BMSCs. The results, shown in Fig. S3, indicated that the isolated and cultured BMSCs were negative for CD45 (0.9%) and CD31 (0.3%). The isolated stem cells uniformly expressed CD44 (99.0%) and CD90 (97.8%), confirming that the cells were indeed BMSCs.

**Fig. 3.**
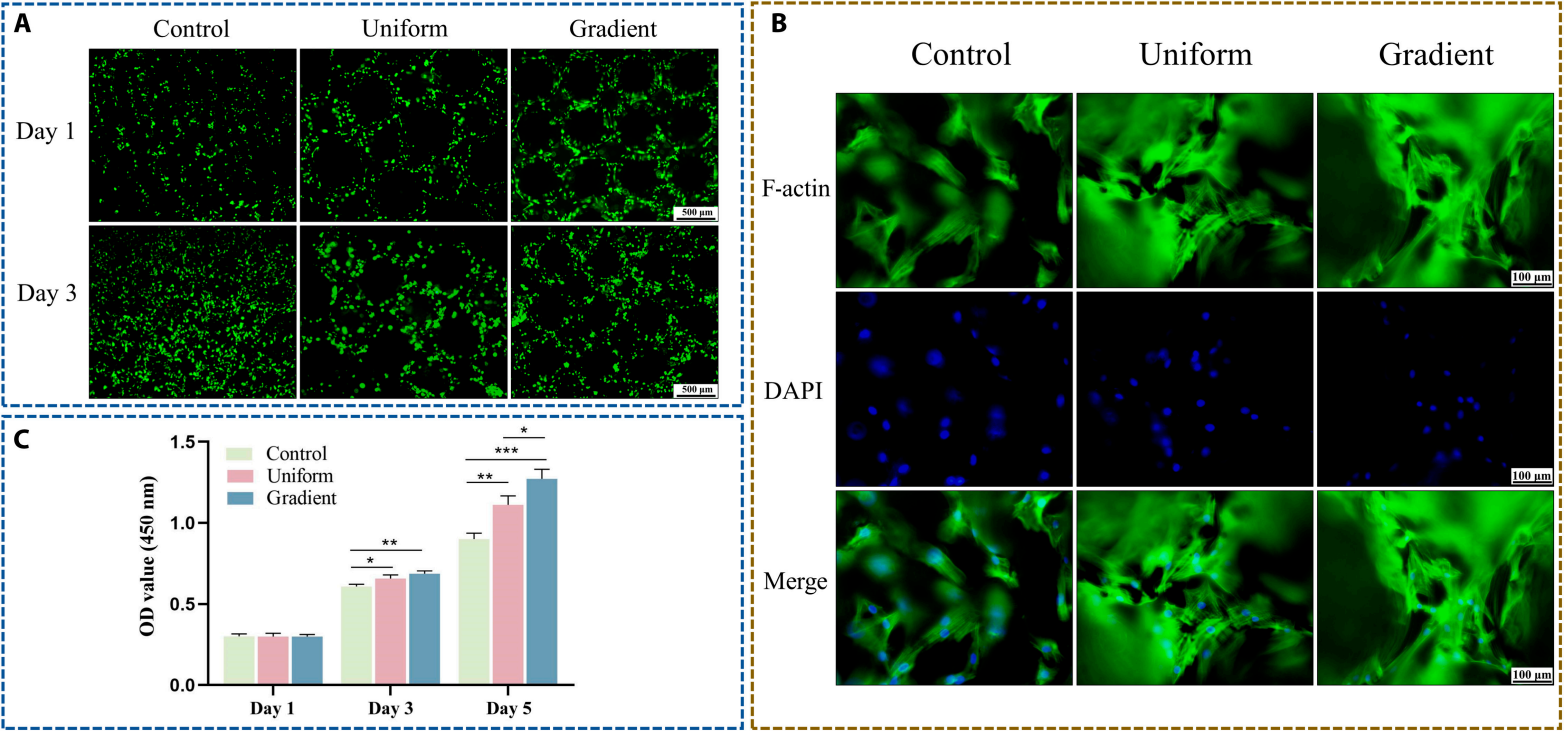
Proliferation activity and biocompatibility of BMSCs on the scaffolds. (A) Live/dead staining images of BMSCs cultured on scaffolds for 1 and 3 d. (B) Representative images of cytoskeletal (F-actin) and nuclear (2-(4-amidinophenyl)-6-indolecarbamidine dihydrochloride [DAPI]) staining of BMSCs after 3 d of culture. (C) Cell Counting Kit-8 (CCK-8) assay results for BMSCs cultured on scaffolds for 1, 3, and 5 d. Data are presented as mean ± SD (*n* = 3). * indicates significant differences (*P < 0.05; **P < 0.01; ***P < 0.001; ****P < 0.0001). OD, optical density.

Live/dead fluorescence staining was used to assess cell viability on the 3 scaffold types, with live cells emitting green fluorescence and dead cells emitting red (Fig. 3A). Over 1 and 3 d of culture, a progressive increase in cell density was observed on all scaffolds, indicating continued cell adhesion and proliferation. The majority of cells remained viable, with minimal cell death, suggesting the favorable biocompatibility of all scaffold types. Notably, the gradient micropore scaffold supported a higher cell density compared to the other groups over time, highlighting its enhanced capacity for cell adhesion and proliferation. Complementary results from the CCK-8 assay further confirmed this trend, showing a time-dependent increase in cell proliferation across all groups. When BMSCs were cultured on the scaffolds for 1, 3, and 5 d, robust proliferative activity was observed, with the gradient micropore scaffold exhibiting a greater proliferative advantage (Fig. 3C).

BMSCs were co-cultured on each scaffold type for 3 d, with F-actin visualized in green using phalloidin and nuclei counterstained in blue with DAPI (Fig. 3B). Cells adhered uniformly across all scaffold surfaces, exhibiting prominent lamellipodia and well-extended cytoskeletons. Compared with cells on the solid control scaffold, cells on the gradient microporous scaffold displayed markedly greater actin filament elongation, larger cytoskeletal areas, and denser intercellular fiber networks.

### Osteogenic differentiation of BMSCs on scaffolds

**Fig. 4.**
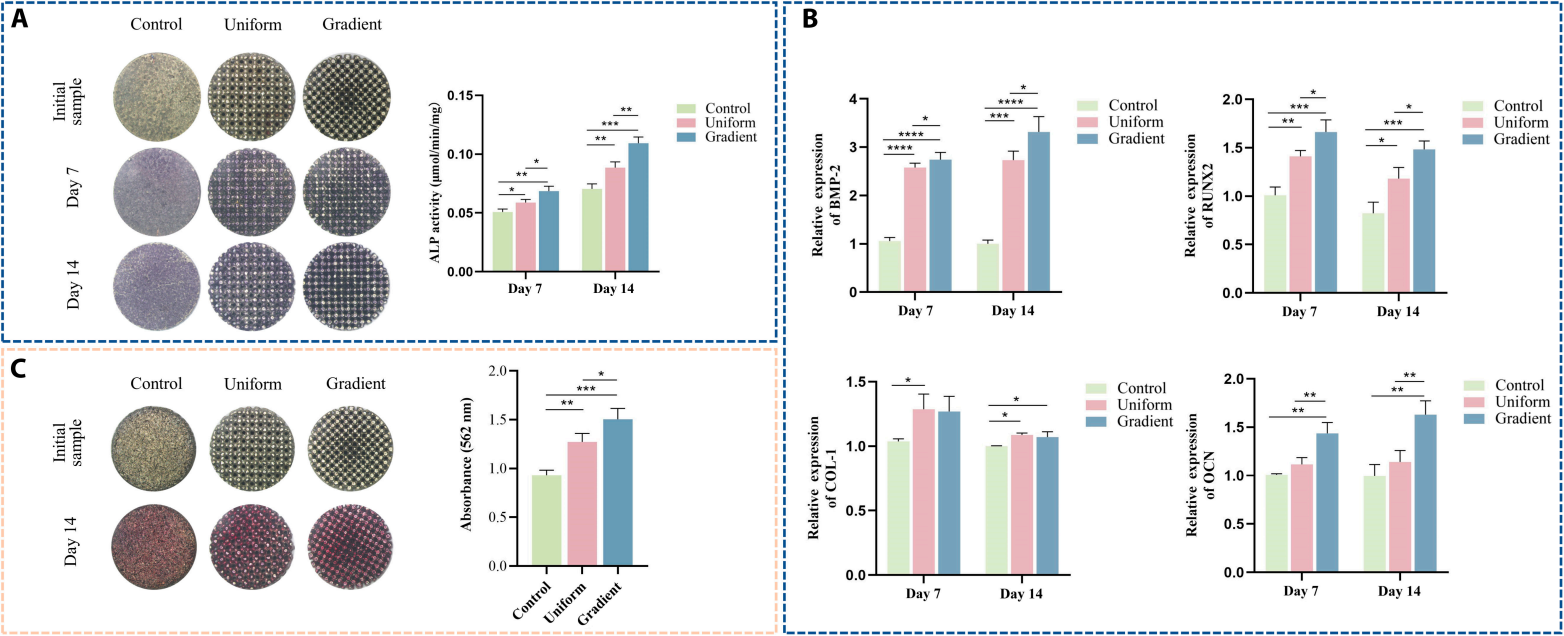
Osteogenic differentiation of BMSCs on different scaffolds. (A) Alkaline phosphatase (ALP) staining images and quantitative analysis after 7 and 14 d. (B) Relative expression levels of osteogenic genes (BMP-2, COL-1, RUNX2, and OCN) after 7 and 14 d. (C) Alizarin Red S (ARS) staining images and quantitative analysis after 14 d. Data are presented as mean ± SD (*n* = 3). * indicates significant differences (*P < 0.05; **P < 0.01; ***P < 0.001; ****P < 0.0001).

Osteogenic differentiation was initially assessed via ALP activity and ARS staining. BMSCs were cultured in osteogenic induction medium for the specified duration to evaluate scaffold effects on osteogenesis. ALP, an early osteoblast differentiation marker, was integral to mineralization [31]. ALP staining at 7 and 14 d revealed blue ALP-positive regions, with deeper color intensity indicating higher positivity (Fig. 4A). All groups exhibited progressive increases in ALP-positive area and staining intensity over time, with the gradient microporous scaffold showing the most pronounced staining. Quantitative ALP analysis corroborated these findings, indicating superior osteogenic potential for the gradient microporous scaffold.

Mineralized nodule formation—a hallmark of terminal osteoblast differentiation—was further assessed by ARS staining at day 14 (Fig. 4C). The gradient microporous scaffold group exhibited more intense staining than the other groups, consistent with ALP activity results.

To further characterize osteoinductive potential, RT-qPCR was performed for BMP-2, RUNX2, COL-1, and OCN. BMP-2 was reported to be essential for bone formation and osteoblast differentiation, regulating ALP activity and the synthesis of COL-1, proteoglycans, fibronectin, and OCN. RUNX2, a key upstream transcription factor, binds osteoblast-specific *cis*-regulatory elements (OSE2) in osteogenic gene promoters and governs early osteogenesis [31–33]. COL-1, secreted by osteoblasts, is the primary fibrous component of the bone matrix, supporting cell adhesion and matrix maturation [34,35]. OCN, expressed during matrix mineralization, functions predominantly in the late phase of osteogenesis [36]. The expression levels of RUNX2 and BMP-2 were significantly higher on microporous scaffolds than on solid controls, with the gradient microporous scaffold outperforming the uniform microporous scaffold (Fig. 4B). Similarly, COL-1 and OCN expression followed the same trend, reflecting enhanced osteogenesis in the gradient microporous group.

Collectively, the gradient microporous scaffold upregulated both early and late osteogenic markers, in agreement with ALP and ARS data. These findings provide robust evidence of the scaffold’s ability to promote osteoblast differentiation and maturation.

### Effect of scaffolds on macrophage biocompatibility and polarization

**Fig. 5.**
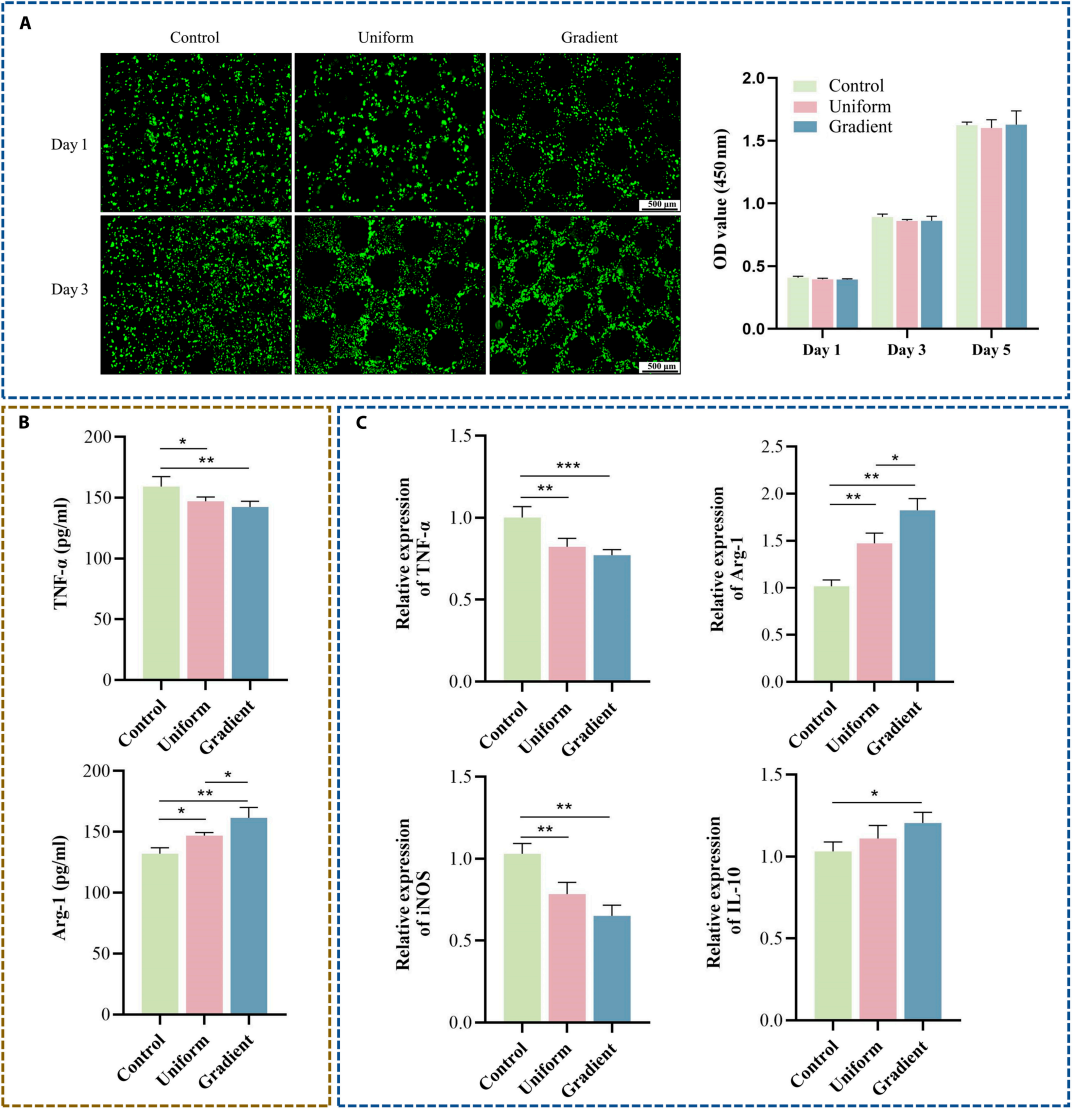
Evaluation of macrophage biocompatibility and polarization. (A) Live/dead staining and CCK-8 assay of Raw 264.7 cells cultured on scaffolds for the indicated times. (B) Enzyme-linked immunosorbent assay (ELISA) measurement of tumor necrosis factor-α (TNF-α) and arginase 1 (Arg-1) levels in macrophage supernatants after 4 d. (C) Real-time quantitative polymerase chain reaction (RT-qPCR) analysis of immune-related gene expression in macrophages cultured on scaffolds for 4 d. Data are presented as mean ± SD (*n* = 3). * indicates significant differences (*P < 0.05; **P < 0.01; ***P < 0.001; ****P < 0.0001). iNOS, inducible nitric oxide synthase; IL-10, interleukin-10.

Raw 264.7 cells exhibited good biocompatibility across all scaffold groups, with cell numbers increasing over time, indicating sustained proliferation (Fig. 5A). No significant intergroup differences in OD values were detected. ELISA analysis quantified TNF-α (M1) and Arg-1 (M2) secretion (Fig. 5B). The solid control scaffold induced the highest TNF-α levels. Although TNF-α expression did not differ significantly between the gradient and uniform microporous groups, the gradient group showed a slight reduction. In contrast, Arg-1 secretion was highest in the gradient microporous group, suggesting a shift toward an anti-inflammatory phenotype.

Following ELISA, RT-qPCR was conducted on scaffold-associated macrophages (Fig. 5C). TNF-α and iNOS served as M1 markers, while IL-10 and Arg-1 were assessed as M2 markers. Both microporous scaffold types showed reduced M1 gene expression compared with the solid control, with no significant differences between the microporous groups. M2-associated genes were upregulated in both microporous groups, with the gradient microporous scaffold eliciting the highest expression. These RT-qPCR results were consistent with ELISA data, confirming that gradient microporous scaffolds promoted M2 polarization while suppressing pro-inflammatory M1 activity.

### The macrophage-conditioned medium induces the osteogenic differentiation of BMSCs

**Fig. 6.**
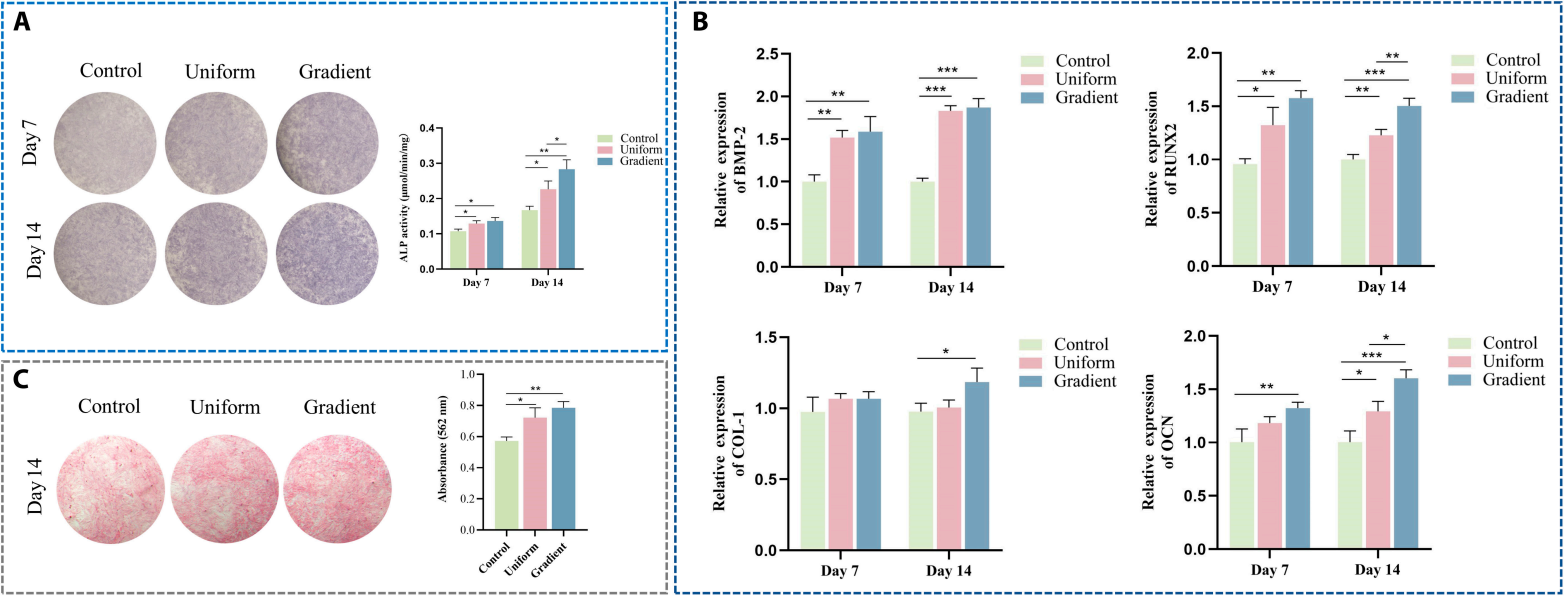
Osteogenic differentiation following co-culture of macrophages and BMSCs. (A) ALP staining images and quantitative analysis after 7 and 14 d of co-culture. (B) RT-qPCR analysis of osteogenesis-related factors (BMP-2, COL-1, RUNX2, and OCN) after 7 and 14 d of co-culture. (C) ARS staining images and quantitative analysis after 14 d of co-culture. Data are presented as mean ± SD (*n* = 3). * indicates significant differences (*P < 0.05; **P < 0.01; ***P < 0.001; ****P < 0.0001).

Macrophages, as key effector cells in bone immunity, interact with osteoblasts to establish an immune microenvironment conducive to osteogenesis upon polarization. To investigate the influence of macrophage polarization on osteogenic differentiation across different scaffold types, BMSCs from each group were cultured in conditioned medium for 7 and 14 d. Osteogenic potential was assessed by ALP activity assays, revealing a time-dependent increase in ALP expression, with the gradient microporous scaffold group exhibiting the highest levels, followed by the uniform microporous scaffold group (Fig. 6A). ARS staining on day 14 demonstrated more intense mineral deposition in the gradient microporous scaffold group (Fig. 6C). Furthermore, RT-qPCR confirmed elevated expression of osteogenic genes—RUNX2, BMP-2, OCN, and COL-1—in the gradient microporous scaffold group (Fig. 6B).

Collectively, these results indicate that the conditioned medium from the gradient microporous scaffold most effectively promoted osteogenic differentiation, suggesting that Raw 264.7 cells cultured on these scaffolds exert superior immune-modulated osteogenic effects.

### Macrophage polarization assessment in the rat subcutaneous implantation model

**Fig. 7.**
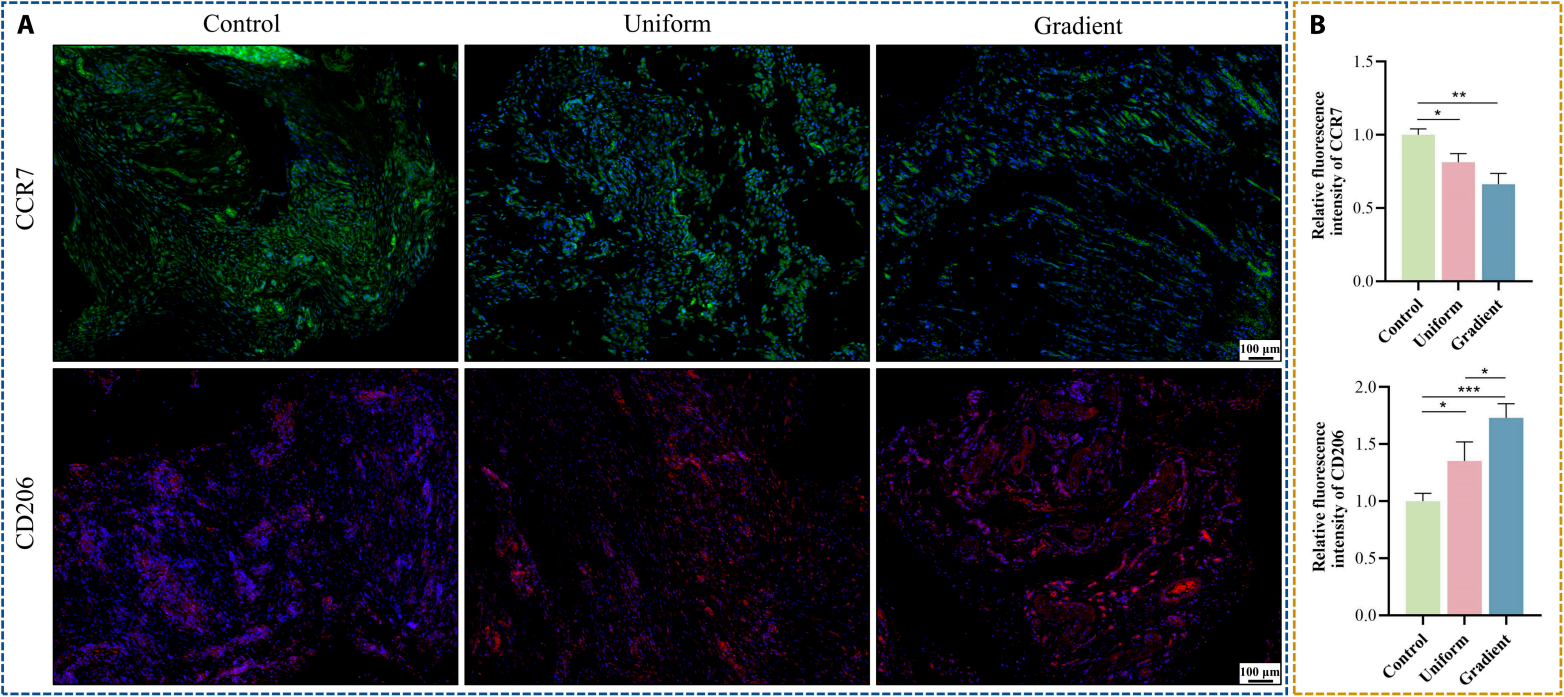
In vivo immune modulation evaluation. (A) Immunofluorescence staining of C-C motif chemokine receptor 7 (CCR7, M1, green) and mannose receptor (CD206, M2, red). (B) Quantitative analysis of CCR7 and CD206 expression. Data are presented as mean ± SD (*n* = 3). * indicates significant differences (*P < 0.05; **P < 0.01; ***P < 0.001; ****P < 0.0001).

To examine in vivo macrophage polarization induced by different scaffolds, 3 scaffold types were implanted subcutaneously in rats and retrieved after 14 d. Immunofluorescence staining of peri-implant tissue revealed higher CCR7 (M2) expression and a lower proportion of iNOS (M1) macrophages in the gradient microporous scaffold group compared to those in the other groups (Fig. 7A and B). These in vivo findings aligned with the in vitro results, demonstrating that the gradient microporous scaffold more effectively induced macrophage polarization, regulated the immune microenvironment, and promoted anti-inflammatory M2 phenotype polarization relative to the uniform microporous and solid control scaffolds.

### X-ray imaging and micro-CT analysis

**Fig. 8.**
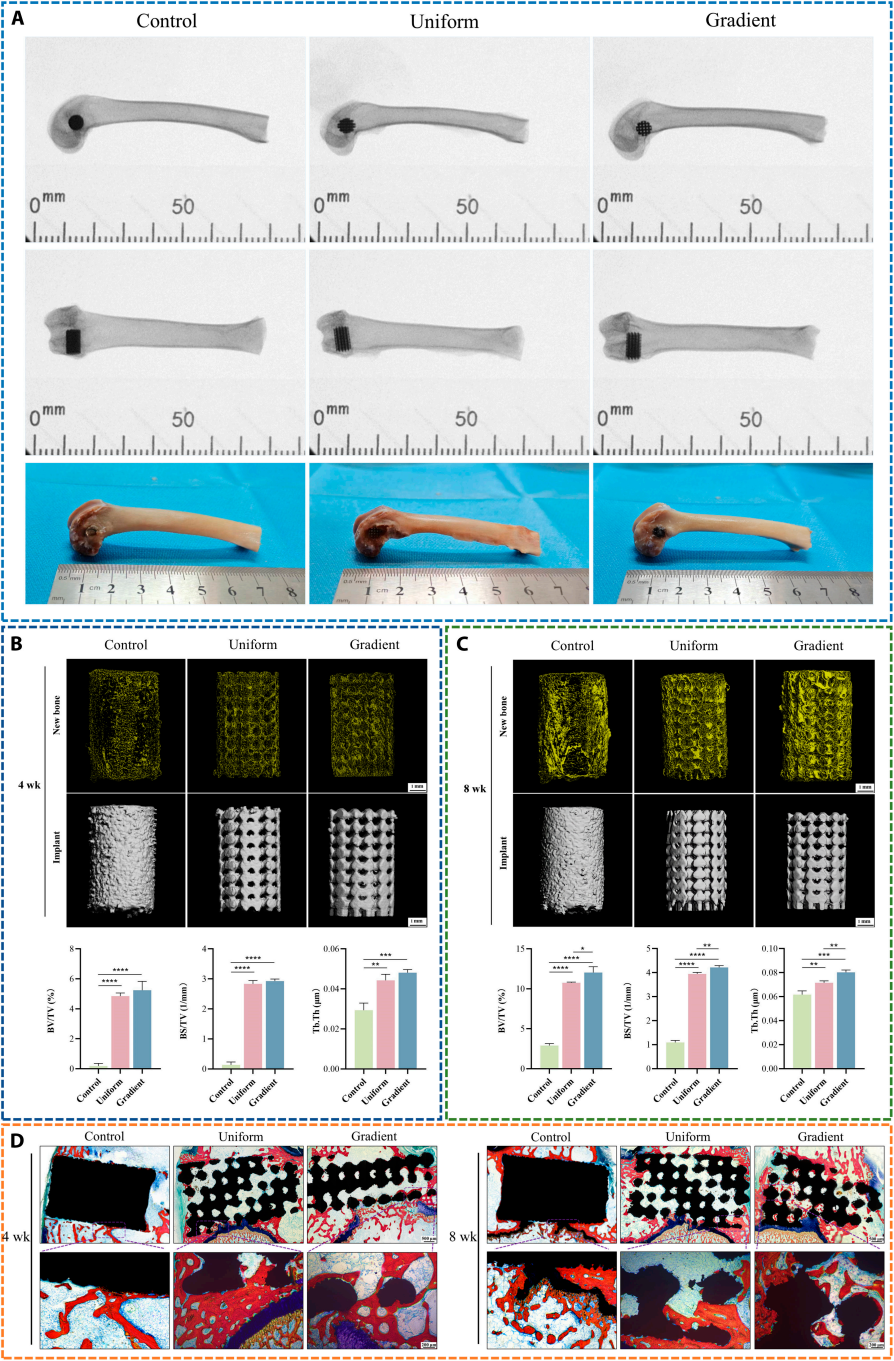
X-ray, micro-computed tomography (micro-CT), and histological images of femoral distal implantation with different scaffold groups. (A) X-ray images and representative specimen images of the femoral distal region after scaffold implantation. (B) Micro-CT images of new bone formation at the defect site 4 weeks postimplantation (yellow represents newly formed bone tissue; gray represents the scaffold), together with quantitative analysis of bone growth parameters. (C) Micro-CT images of new bone formation at the defect site 8 weeks postimplantation, together with quantitative analysis of bone growth parameters. (D) Methylene blue–acid fuchsin staining of sections at 4 and 8 weeks for different scaffold groups (red and dark red represent mineralized and newly formed bone, respectively, and black represents the scaffold). Data are presented as mean ± SD (*n* = 3). * indicates significant differences (*P < 0.05; **P < 0.01; ***P < 0.001; ****P < 0.0001). BV, bone volume; TV, total volume; BS, bone surface area; Tb.Th, trabecular thickness.

Three types of scaffolds were implanted into distal femoral condyle defects in rabbits to evaluate their in vivo osteogenic performance. Radiographic evidence was used to assess new bone formation and distribution within the scaffolds. Quantitative analysis was performed to demonstrate differences in bone growth potential among the scaffolds. X-ray images and photographs of the rabbit femur samples with implanted scaffolds are shown in Fig. 8A. The results indicate that all scaffolds were successfully implanted at the designated sites, with no signs of loosening or displacement.

Micro-CT was used to assess new bone formation in different scaffold groups at 4 and 8 weeks (Fig. 8B and C). In the images, gray represents the scaffold, and yellow indicates new bone. The gradient microporous scaffold group demonstrated superior new bone formation at both 4 and 8 weeks compared to the other 2 groups. Further analysis was conducted using bone parameters, including BV/TV, BS/TV, and Tb.Th. BV/TV and BS/TV directly and indirectly reflect changes in bone volume. At week 8, the BS/TV results for the gradient microporous scaffold group showed significantly higher new bone formation compared to the other 2 groups, with the BV/TV data also indicating a clear promotion of bone regeneration by the gradient microporous scaffold. Tb.Th is primarily used to assess the impact of scaffolds, particularly microporous scaffolds, on trabecular thickness. A higher Tb.Th value correlates with better trabecular quality. Over time, the gradient microporous scaffold group also exhibited a significant increase in Tb.Th.

### Histological section analysis

Histological evaluation was conducted using methylene blue–acid fuchsin staining of coronal sections at 4 and 8 weeks (Fig. 8D). In these images, mineralized bone appears red, newly formed bone appears deep red, and the scaffold material appears black. Newly formed bone and mineralization were observed around all scaffolds. In the solid control group, the absence of internal pores restricted bone formation to the scaffold’s outer surface, with a gradual increase over time but a distinct boundary between the bone and the scaffold. In contrast, the microporous designs of the uniform and gradient scaffolds enabled progressive ingrowth of mineralized and newly formed bone toward the scaffold interior. This effect was most pronounced in the gradient microporous group, where deeper and more extensive bone penetration was observed, indicating superior osseointegration compared with that of both the uniform microporous and solid control scaffolds.

### Macrophage polarization and bone regeneration around scaffolds in vivo

**Fig. 9.**
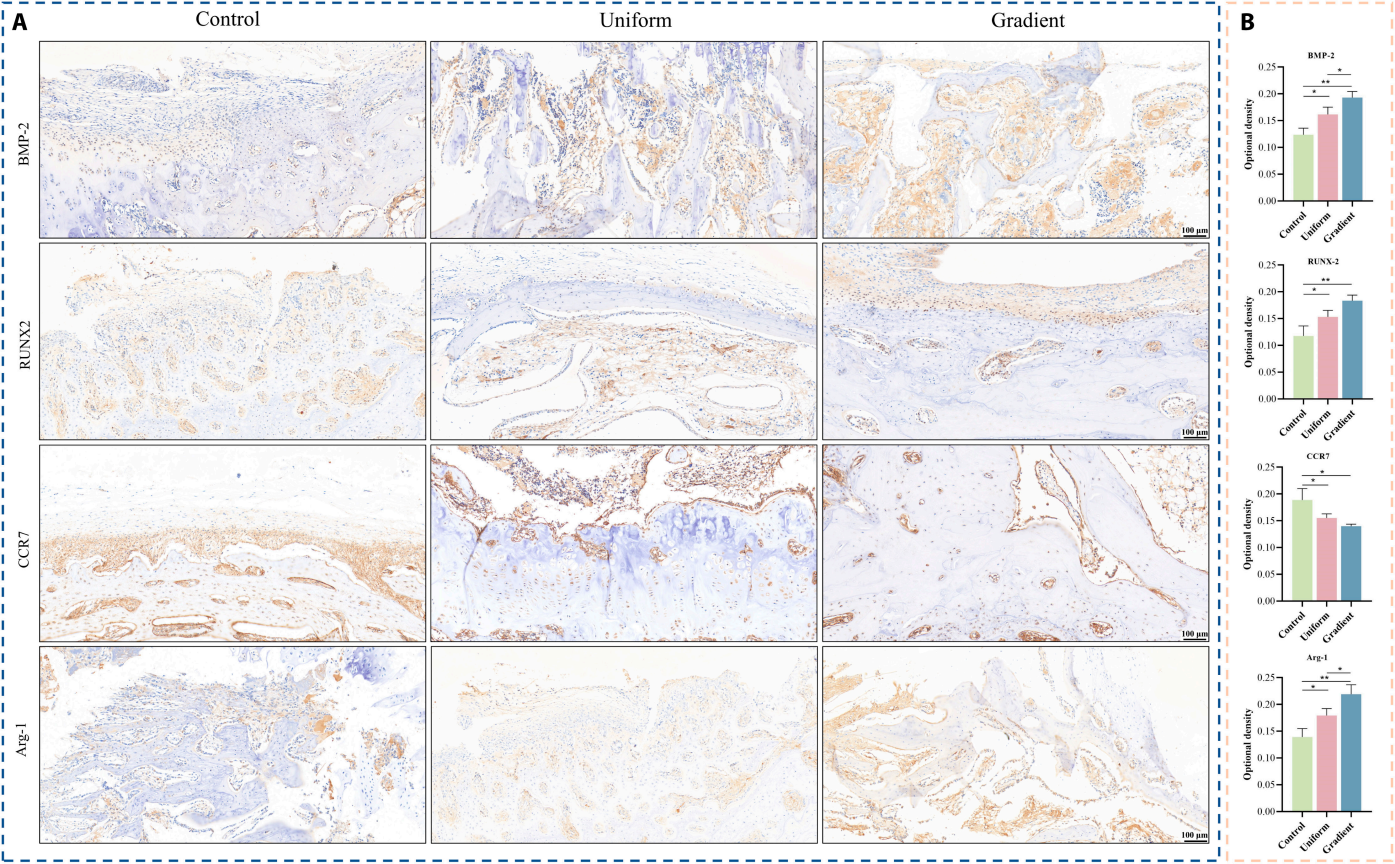
Immunohistochemistry (IHC) staining images of bone tissue surrounding the scaffolds after 4 weeks of implantation. (A) IHC staining images of osteogenesis-related markers (BMP-2 and RUNX2) and macrophage polarization markers (CCR7 [M1] and Arg-1 [M2]). (B) Quantitative analysis of various markers from IHC staining images. Data are presented as mean ± SD (*n* = 3). * indicates significant differences (*P < 0.05; **P < 0.01; ***P < 0.001; ****P < 0.0001).

To evaluate the polarization state of macrophages surrounding the implants and their effect on adjacent newly formed bone tissue, scaffolds implanted in rabbits for 4 weeks were gradually decalcified. Following removal of the implants from the bone defect sites, IHC staining was performed on the surrounding bone tissue (Fig. 9A and B). The osteogenesis-related marker BMP-2 exhibited higher positive expression in the gradient microporous scaffold group than in the other 2 groups, and RUNX2 expression was also significantly elevated in this group. Additionally, the macrophage polarization markers CCR7 (M1) and Arg-1 (M2) were assessed. Both were positively expressed in the peri-implant bone tissue; however, the gradient microporous scaffold group demonstrated a relatively lower proportion of M1 macrophages and a higher proportion of M2 macrophages compared with the other groups. These findings suggest that enhanced M2 macrophage polarization may play a pivotal role in promoting osteogenesis in the gradient microporous scaffold group.

## Discussion

This investigation focuses on the immune and osseointegration responses elicited by TPMS-P-based biomimetic gradient microporous scaffolds. Research has shown that the implantation of biomaterials into the host body often triggers immune responses, with macrophages being one of the most crucial regulatory and effector cells in the immune microenvironment. Macrophages have been widely used to evaluate the immunomodulatory properties of biomaterials [17,37]. While some studies have explored the impact of microporous structures on macrophage polarization, most of the research has focused on the effects of uniform microporous structures on bone immune modulation [7,20,21,35]. To date, no studies have reported the effects of TPMS-P-based biomimetic gradient microporous scaffolds on bone immunomodulation. Therefore, in this study, biomimetic gradient microporous scaffolds were designed based on this structure and fabricated using 3D printing. Both in vitro and in vivo experiments demonstrated that this scaffold configuration was more favorable for modulating immune responses and for promoting osteogenesis and osseointegration.

SEM revealed that all 3 types of scaffolds exhibited partially melted Ti6Al4V particles generated during the SLM process, resulting in surfaces that were not completely smooth. Previous studies have indicated that a moderate level of roughness on the scaffold surface can effectively increase its surface area, providing more sites for protein adsorption. This enhances cell extension, adhesion, proliferation, and the favorable integration of bone tissue with the scaffold [38]. In terms of mechanical properties, compression tests were performed on the scaffolds to determine their elastic modulus. Existing research suggests that if the elastic modulus of an implant does not match that of the bone tissue, it may lead to stress shielding effects, causing local bone resorption and fractures [27]. In this study, compared to the high elastic modulus of the solid scaffold, the elastic moduli of the uniform microporous scaffold and gradient microporous scaffold were significantly reduced, falling within the range of that of cortical bone (3 to 30 GPa). This reduction in modulus may alleviate the stress shielding effect [22]. Further observations revealed that the elastic modulus of the gradient microporous scaffold was slightly lower than that of the uniform microporous scaffold. It is known that porosity and pore size are inversely related to mechanical properties. The presence of relatively larger pores in certain regions of the gradient microporous scaffold, compared to those in the uniform microporous scaffold, may contribute to its lower mechanical strength. This phenomenon is consistent with the findings of previous studies [15,16]. Although these scaffolds demonstrate promising biological and mechanical properties, long-term performance under cyclic or sustained mechanical loading remains untested. Fatigue and durability assessments are essential to evaluate structural stability and biocompatibility over extended implantation periods. Future research will address these aspects to ensure the clinical reliability and adaptability of gradient microporous scaffolds under diverse biomechanical conditions.

In vitro results demonstrated that all 3 scaffolds exhibited good cell proliferation and adhesion capabilities, confirming their biocompatibility and nontoxicity. CCK-8 assays and live/dead staining demonstrated that the gradient microporous scaffold exhibited superior cell proliferation and viability compared with the other groups. In contrast, cells on the solid control scaffold were restricted to surface growth due to the absence of internal void space. The enhanced proliferation on the gradient microporous scaffold can be explained by several synergistic mechanisms. The smaller pore regions promote extracellular matrix (ECM) deposition, cell adhesion, and differentiation, whereas larger pores facilitate nutrient diffusion, cell migration, and ion exchange while providing a sufficient volume for ECM accumulation [15,16]. This hierarchical pore arrangement prevents cell congestion and simultaneously enhances angiogenesis and nutrient transport. Furthermore, the TPMS-based architecture of the gradient microporous scaffold ensures exceptional pore connectivity, high permeability, and an elevated specific surface area, thereby maximizing the available surface for cell attachment and proliferation [39]. Phalloidin and DAPI dual-staining images after 3 d of culture on different scaffolds revealed that cells on the gradient microporous scaffold exhibited more elongated F-actin extensions and a denser, more interconnected cytoskeletal network. This phenomenon is likely attributed to the pore size distribution of the gradient microporous scaffold: the combination of multiple pore sizes provides diverse topographical cues for cell adhesion and migration, facilitating F-actin polymerization and the extension of lamellipodia and filopodia [12,40]. Additionally, curvature variations inherent to the TPMS geometry may further stimulate tissue regeneration, as cells are known to sense local curvature and adapt their morphology accordingly. This mechanotransductive feedback can drive actin fiber remodeling and membrane protrusion extension, thereby enhancing the scaffold’s bioactivity [39,40].

In osteogenic differentiation assays, the gradient microporous scaffold demonstrated a markedly higher ALP activity, more intense ARS staining, and elevated osteogenic gene expression relative to controls. Several factors contribute to these effects. First, the scaffold’s pore size and geometry enhance permeability, which supports osteoblast proliferation, metabolic activity, and bone tissue regeneration [41]. Second, the concave and convex surfaces of the TPMS gradient structure provide more physiologically relevant curvature cues than flat grid designs, facilitating osteogenic differentiation within optimal curvature ranges [39]. Third, the high surface-to-volume ratio increases opportunities for cell–protein interactions, fostering adhesion, proliferation, and binding with bioactive molecules such as growth factors and ECM proteins, thereby amplifying osteoinductive potential [42]. In vivo implantation further confirmed these advantages: the microporous architecture promoted strong interfacial bonding between the implant and host bone, accelerating integration and healing. The TPMS-based gradient microporous scaffold achieved greater bone ingrowth and deeper new bone formation compared with uniform-pore scaffolds. These findings align with the work of Li et al. [27], who reported that gradient microporous titanium alloy scaffolds with TPMS-P architecture exhibited significantly superior osteogenic performance over solid scaffolds in a pig tibia implantation model.

Although numerous studies have demonstrated the strong osteogenic potential of microporous scaffolds in repairing bone defects, the immune responses induced by the implantation of these scaffolds, such as chronic inflammation, fibrosis, and implant failure, have become a growing concern [11,16,23]. Evidence indicates that these immune reactions are closely linked to protein–material interactions. Protein type, concentration, and adsorption mode vary with the material’s morphology, which in turn influences protein conformation and immune cell activity [43]. Among morphological parameters, pore size is particularly important, as it modulates immune cell behavior—especially macrophages—by directing their polarization toward either a pro-inflammatory (M1) or an anti-inflammatory (M2) phenotype. M1 macrophages exacerbate local inflammation via the secretion of pro-inflammatory cytokines, including IL-1β, IL-6, IL-12, IL-23, TNF-α, and iNOS, whereas M2 macrophages facilitate tissue repair and bone homeostasis through anti-inflammatory mediators such as IL-10, Arg-1, and transforming growth factor-β [18,44].

In this study, cell proliferation assays confirmed that all 3 scaffold types exhibited excellent biocompatibility. Using these scaffolds as model systems, the effects of structural variation on macrophage behavior were systematically examined in vitro. TNF-α, a key pro-inflammatory cytokine, plays a dual role: while it can promote tissue repair and regeneration in certain contexts, excessive levels impede healing by preventing the transition of inflammatory macrophages to the repair-oriented phenotype [45]. IL-10, a potent anti-inflammatory cytokine, is critical for inducing M1-to-M2 phenotypic switching [36]. Additionally, iNOS and Arg-1 act as enzymatic markers of macrophage polarization: iNOS typically amplifies pro-inflammatory responses, whereas Arg-1, by catalyzing l-arginine, exerts anti-inflammatory effects and influences mitochondrial oxidative phosphorylation, thereby contributing to bone homeostasis [32,33]. Furthermore, the transcriptional level of Arg-1 can influence energy metabolism by regulating mitochondrial oxidative phosphorylation, thereby affecting bone homeostasis [33]. To characterize these responses, ELISA and RT-qPCR were employed to quantify M1 markers (TNF-α and iNOS) and M2 markers (IL-10 and Arg-1) in Raw 264.7 macrophages. Results revealed that the gradient microporous scaffold displayed superior anti-inflammatory activity compared with solid and uniform microporous controls. These findings suggest that scaffold surface morphology directs macrophage polarization, leading to the secretion of distinct cytokine profiles that shape the immune microenvironment and promote tissue regeneration [33]. Notably, several macrophage-derived mediators synergize with other microenvironmental cues to create conditions favorable for bone formation. Prior work has shown that mesenchymal stem cells exhibit higher survival rates when co-cultured with M2 macrophages than with M1 macrophages [34]. Moreover, macrophage-conditioned media from microporous scaffolds can significantly enhance the osteogenic differentiation of BMSCs in vitro [35]. Accordingly, to evaluate the impact of macrophage polarization on osteogenesis, this study employed indirect co-culture of macrophages and BMSCs to assess the expression of osteogenic markers—such as ALP, ARS, and osteogenesis-related genes—in BMSCs influenced by Raw 264.7 macrophages cultured on the different scaffolds. Our results indicate that the gradient microporous scaffold exhibited superior osteogenic ability, suggesting that the biomimetic gradient microporous structure can better regulate the immune microenvironment to enhance bone formation.

**Fig. 10.**
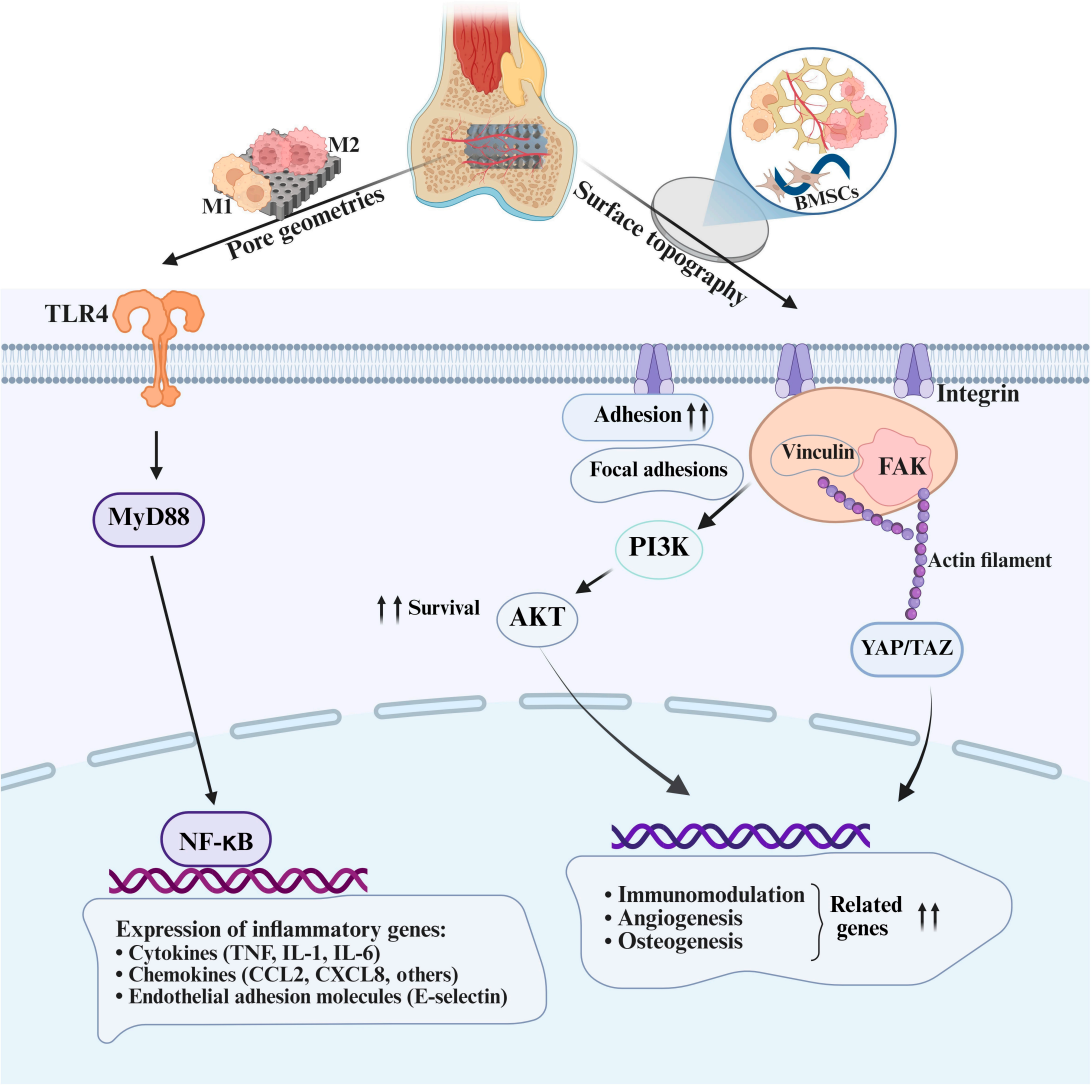
Schematic representation of the potential mechanism underlying bone immunomodulation by biomimetic gradient microporous scaffolds. This diagram illustrates the interactions between the biomimetic gradient microporous scaffold, immune response, and osteogenesis. It highlights the involvement of the TLR4/MyD88/NF-κB signaling pathway, focal adhesion kinase (FAK) pathway, and their downstream AKT and yes-associated-protein (YAP) pathways, ultimately leading to macrophage polarization and subsequent bone integration. The figure was created with Biorender.com. PI3K, phosphoinositide 3-kinase; TAZ, transcriptional coactivator with PDZ-binding motif; CCL2, C-C motif ligand 2; CXCL8, C-X-C motif chemokine ligand 8.

In the in vivo subcutaneous implantation model, the gradient microporous scaffold exhibited a higher proportion of M2 macrophage infiltration. Further immunohistochemical analysis of the bone tissue surrounding the gradient microporous scaffold revealed that the TPMS-based biomimetic gradient microporous titanium alloy scaffold was more conducive to promoting macrophage polarization toward the M2 phenotype. At the same time, osteogenesis-related markers in the surrounding microenvironment showed an upregulation trend, suggesting a potential enhancement in the osteogenic differentiation potential of BMSCs. This observation may be attributed to the unique features and morphological characteristics of the biomimetic gradient microporous structure (Fig. 10), such as pore size, porosity, and curvature, which provide extracellular microenvironmental cues that create a favorable immune microenvironment for cells. On the one hand, biomimetic gradient microporous scaffolds with appropriate porosity and gradient porous structures support the growth and differentiation of stem cells, as well as the adhesion and activation of macrophages, which are critical for promoting macrophage polarization and subsequent osseointegration. Multiple intracellular signaling pathways are believed to play a role in regulating macrophage polarization. For example, myeloid differentiation primary response protein 88 (MyD88) expression—responsive to variations in pore size—plays a central role in the canonical toll-like receptor 4 (TLR4)/MyD88/nuclear factor-κB (NF-κB) signaling axis, which substantially modulates macrophage phenotype transformation, although it is not the sole determining factor [21,46]. Thus, gradient microporous scaffolds may modulate immune responses through TLR4/MyD88/NF-κB signaling, thereby enhancing osseointegration. At the same time, the surface curvature of the gradient microporous scaffold facilitates the proliferation and differentiation of osteoblasts, with growth factors and cytokines tending to deposit on the concave surfaces of the scaffold rather than other surfaces. This results in a higher concentration of cytokines in these regions. The geometric shape of the pores can influence the initial inflammation surrounding the scaffold, with larger pore sizes and concave surfaces potentially inducing higher levels of pro-inflammatory cytokine secretion during the early stages of bone regeneration, which may facilitate bone tissue regeneration [16,47]. Furthermore, studies have suggested that the design of a layered interconnected porous structure can improve the paracrine function of mesenchymal stem cells cultured on the scaffold by affecting 2 pathways related to ECM and mechanotransduction, namely, the FAK pathway and the downstream AKT and YAP pathways. This, in turn, regulates the local immune microenvironment, promotes M2 macrophage polarization, and induces osteogenesis and bone regeneration both in vitro and in vivo [48,49]. Despite these promising results, the precise molecular mechanisms by which biomimetic gradient microporous scaffolds coordinate inflammation and regeneration remain incompletely understood. Future research will focus on elucidating these pathways to optimize scaffold design for enhanced immunomodulation and bone regeneration.

However, there are several limitations in this study. First, to closely simulate clinical applications and avoid the potential impact of scaffold degradation on micropore structure size, titanium alloy was chosen as the material. However, the rigidity of metal materials limits the accuracy of experimental results, such as IHC analysis, within the scaffold. Future studies should consider the use of materials such as hydroxyapatite or polycaprolactone for in vivo evaluations. Second, this study only explored the advantages of a single type of gradient microporous scaffold over uniform microporous scaffolds and solid scaffolds in terms of bone immune modulation. Therefore, future research should design scaffolds with a broader range of gradient structures to identify the optimal pore size gradient and investigate its effects on the bone immune microenvironment, as well as its underlying mechanisms. Finally, the in vivo experimental duration was relatively short. Extended preclinical studies are warranted to evaluate the long-term implantation performance of these scaffolds, thereby validating their safety, reliability, and efficacy in bone tissue repair and regeneration. Such investigations will facilitate optimization of implant design and accelerate clinical translation.

## Conclusion

This study fabricated titanium alloy biomimetic gradient microporous scaffolds with a TPMS structure using SLM technology. Both in vitro and in vivo results confirmed that these scaffolds exhibited excellent osseointegration. Furthermore, the gradient microporous architecture modulated immune responses, synergistically promoting osteogenesis and enhancing osseointegration. These results highlight the scaffolds’ strong translational potential and provide a theoretical framework for future design of gradient microporous prosthetic structures.

## Data Availability

The data are freely available upon request.
