# Supplementary material for: Biomimetic Gradient Microporous Scaffold with a Triply Periodic Minimal Surface Enhances Osseointegration by Modulating Macrophage Polarization
Source: Biomater Res. 2025 Oct 17;29:0266. doi: 10.34133/bmr.0266 (PMC12531630; doi:10.34133/bmr.0266)
Supplement: Supplementary 1 — Figs. S1 to S3 Tables S1 and S2 [file bmr.0266.f1.doc]

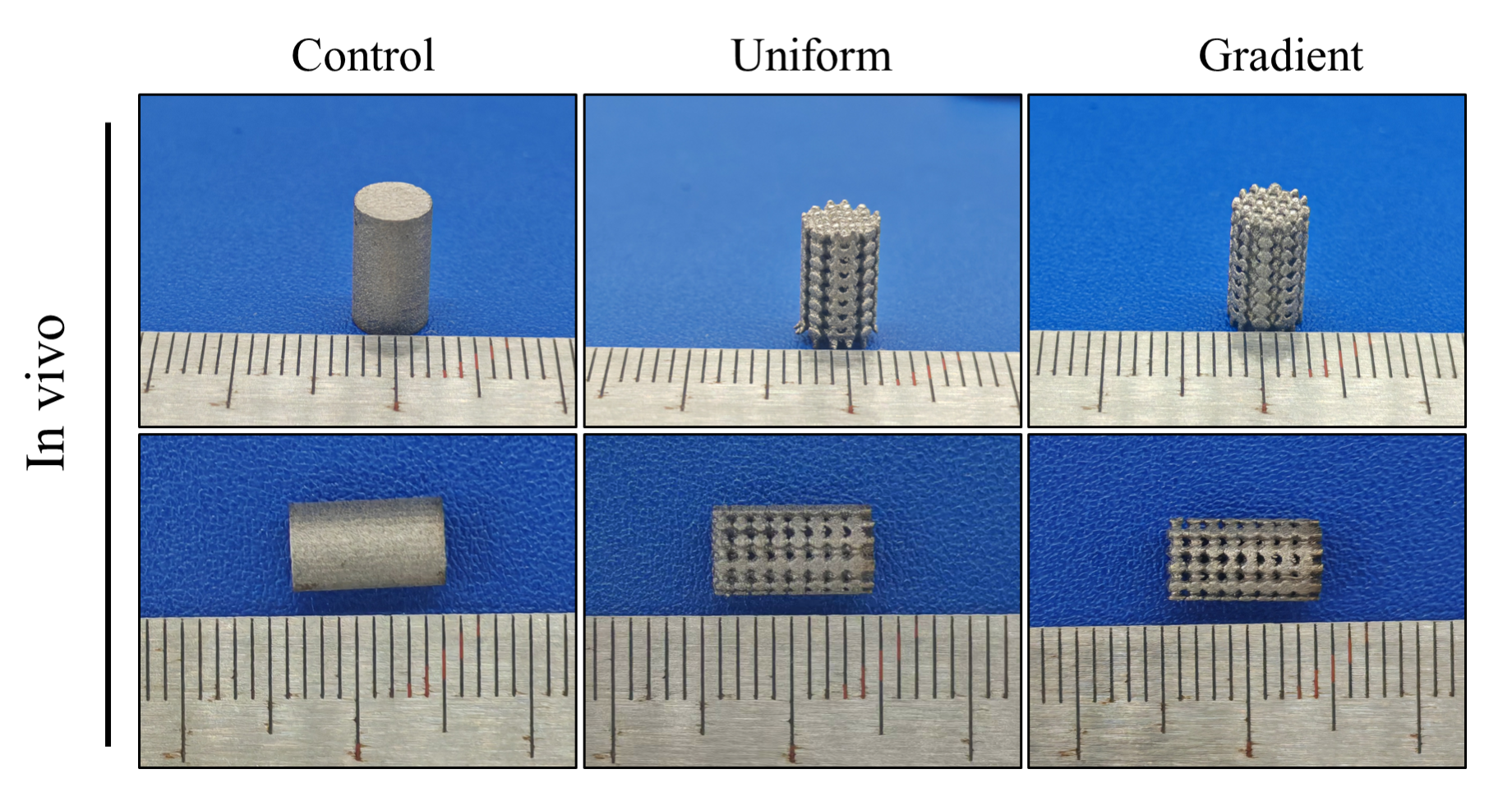
**Fig. S1.** Macroscopic images of different in vivo scaffolds.


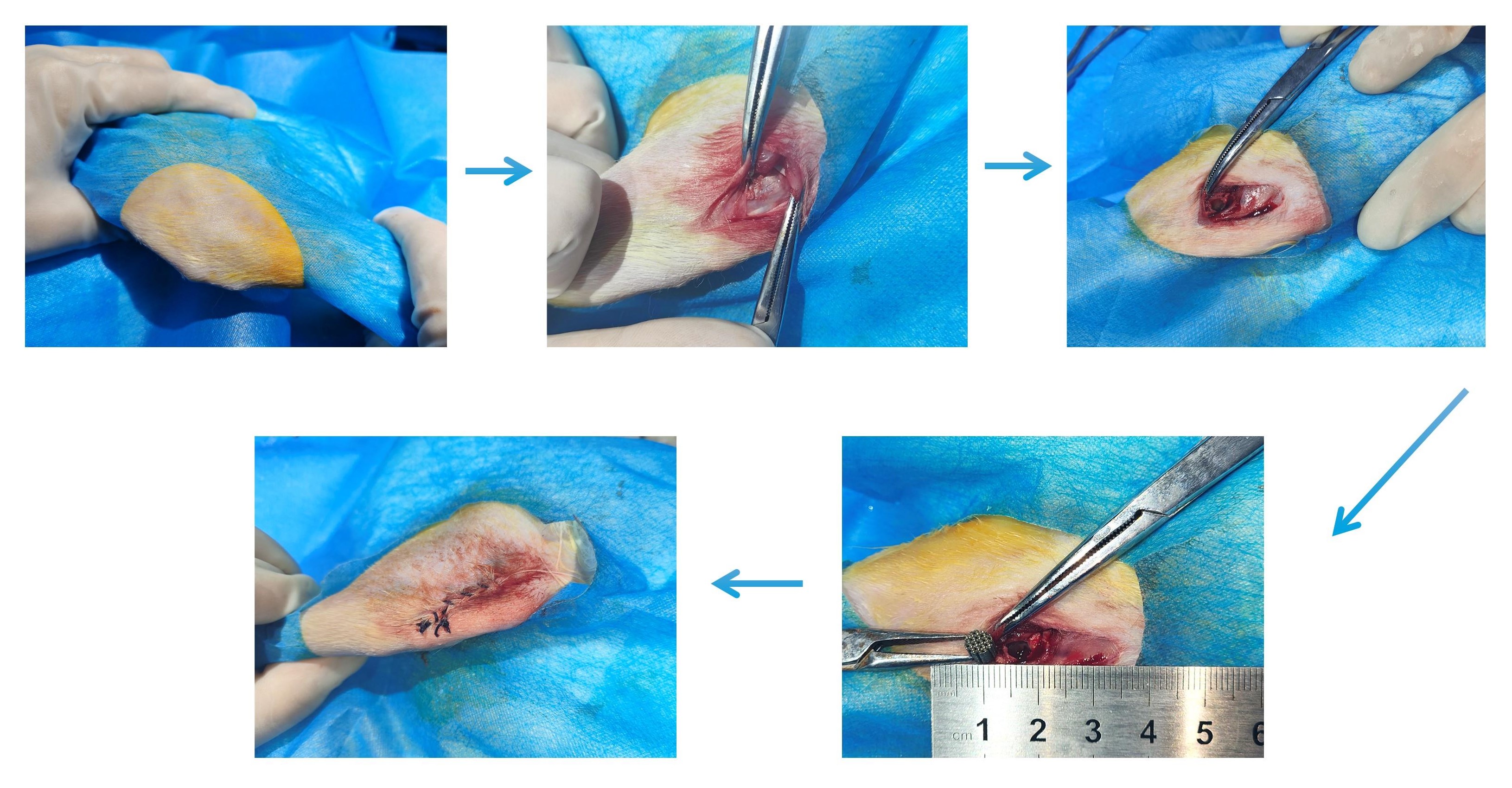


**Fig. S2.** Schematic diagram of in vivo scaffold implantation process.


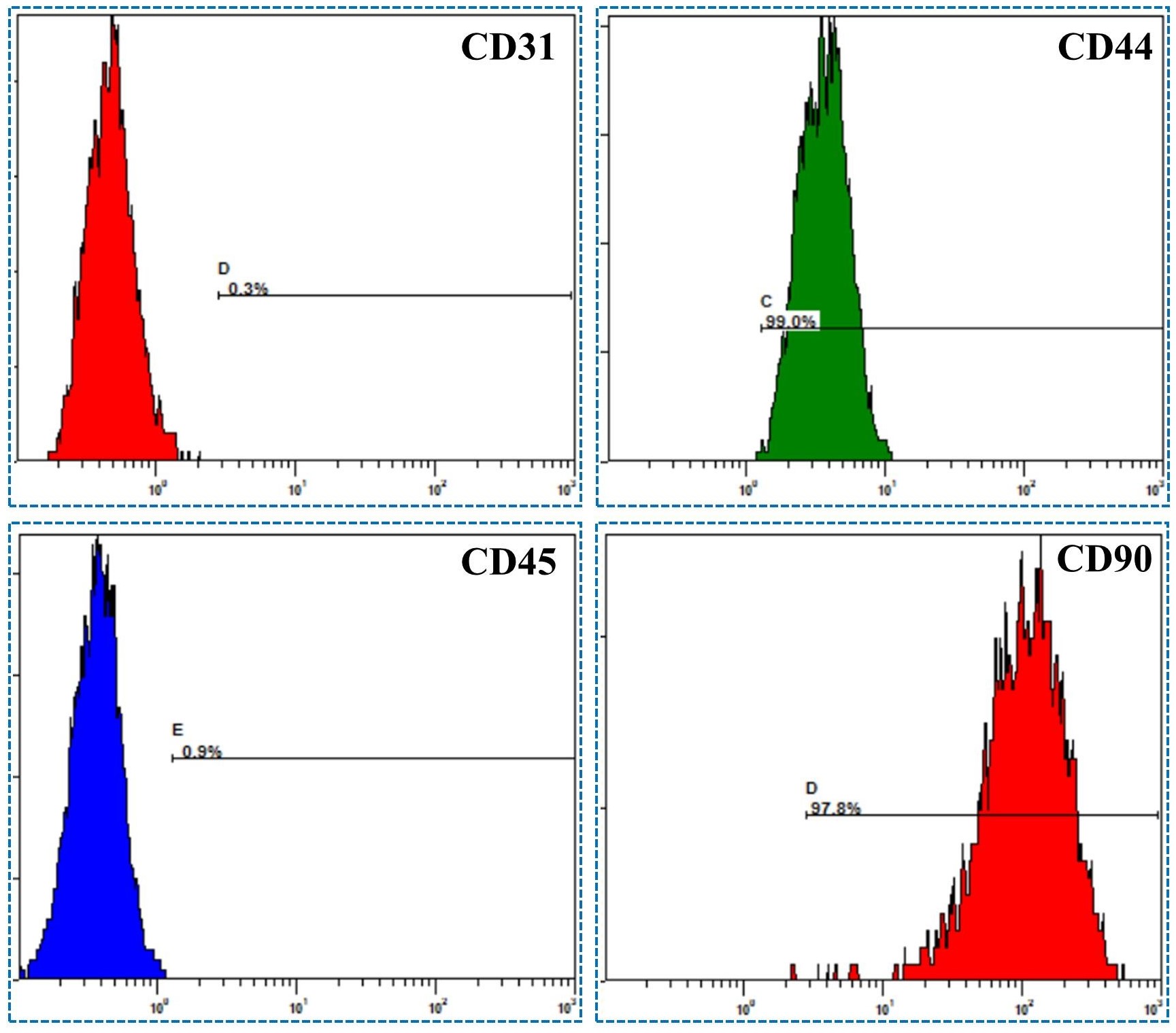


**Fig. S3.** Immunophenotypic analysis of BMSCs by flow cytometry.

**Table S1. Osteogenic Primer Sequences Used in RT-qPCR**

| **Gene** | **Forward primer sequence** | **Reverse primer sequence** |
| --- | --- | --- |
| BMP-2 | GGACCCGCTGTCTTCTAGTG | ACTCAAACTCGCTGAGGACG |
| RUNX2 | TTTCAGACCCCAGGCAGTTC | CGTGTGGTAGTGAGTGGTGG |
| OCN | CCTGACTGCATTCTGCCTCT | CCACCTTACTGCCCTCCTG |
| COL-I | CTGGCAACCTCAAGAAGTCC | CAAGTTCCGGTGTGACTCG |
| GAPDH | CCTCGTCTCATAGACAAGATGGT | GGGTAGAGTCATACTGGAACATG |

**Table S1. Immune primer sequences used in RT-qPCR**

| **Gene** | **Forward primer sequence** | **Reverse primer sequence** |
| --- | --- | --- |
| TNF-α | AGCCGATGGGTTGTACCTTG | ATAGCAAATCGGCTGACGGT |
| iNOS | AGACCTCAACAGAGCCCTCA | TCGAAGGTGAGCTGAACGAG |
| Arg-1 | GCTTGCGAGACGTAGACCCT | CCATCACCTTGCCAATCCC |
| IL-10 | ACCTGGTAGAAGTGATGCCC | CCACTGCCTTGCTCTTATTTT |
| GAPDH | TGACCACAGTCCATGCCATC | GACGGACACATTGGGGGTAG |
